# The potential efficacy of continuous plantar foot cooling during relatively mild simulated occupational heat stress—a pilot study

**DOI:** 10.1007/s00421-025-06053-0

**Published:** 2025-11-18

**Authors:** Jason M. Keeler, Charlie Walker, Amanda Regester, Molly Heikkinen, Blair D. Johnson, Y. Sungtaek Ju, Lihua Jin, Zachary J. Schlader

**Affiliations:** 1 Department of Health and Human Performance, Congdon School of Health Sciences, High Point University, High Point, NC USA; 2 Department of Kinesiology, Indiana University School of Public Health, Bloomington, IN USA; 3 Nutrition and Exercise Research Center, Indiana University School of Public Health, Bloomington, IN USA; 4 Department of Mechanical and Aerospace Engineering, University of California, Los Angeles, CA USA; 5 Department of Bioengineering, University of California, Los Angeles, CA USA

**Keywords:** Heat stress, Foot cooling, Core temperature, Work capacity

## Abstract

**Purpose:**

To identify the effect size regarding glabrous skin foot cooling on core temperature, cognitive performance, and physical work capacity (PWC) during mild simulated occupational heat stress.

**Methods:**

A pilot sample of twelve healthy adults (5 women) completed experimental visits in a 38.7 ± 0.7 ℃ and 46 ± 9% relative humidity environment that consisted of 60-minutes of steady-state upper body ergometry exercise followed by a 60-minute PWC test involving upper body ergometry while maintaining a rating of perceived exertion of 13 (“somewhat hard”). In one trial, participants stood with bare feet on cooled plates (17.7 ± 0.5 ℃) and in the other they were on noncooled plates (36.5 ± 0.1 ℃), with the order assigned in a block randomized manner.

**Results:**

Core temperature increased during steady-state exercise (from 37.5 ± 0.3 to 37.6 ± 0.3 ℃, *p* < 0.003) and the PWC test (from 37.6 ± 0.3 to 37.9 ± 0.3 ℃, *p* < 0.001), but there was no difference between conditions (mean difference 0.03±0.18 ℃, *p* ≥ 0.592, d_z_=0.112). During the PWC test, there was no difference between conditions for mean power output (*p* = 0.213, d_z_=0.184), with the final power outputs of 28 ± 9 W and 30 ± 9 W for control and cooling conditions, respectively. There were no condition effects for cognitive performance on any measure (*p* ≥ 0.207, d_z_≤0.405). However, foot cooling (+ 15 ± 9 bpm) reduced increases of heart rate during the PWC test compared to control (+ 21 ± 11 bpm, *p* = 0.013, d_z_=0.425).

**Conclusion:**

Glabrous skin foot cooling has a relatively small effect on core temperature and PWC, but moderate effects on cognitive performance and heart rate during short duration (~ 3 h) mild heat stress.

## Introduction

While advancements in automation continue, human workers are still expected to play vital roles in many occupations, such as construction, where they are frequently exposed to heat stress (Flouris et al. 2018; Chapman et al. 2021). Within the United States 30–40 million workers encounter heat stress, defined as the net heat load to which a person is exposed, in the workplace (2023a), a number that is likely to increase, as global temperatures rise (US EPA 2021). Heat stress increases the likelihood of hyperthermia, defined as a rise in core (internal) body temperature (Casa et al. 2015; Cramer and Jay 2016). In turn, hyperthermia increases the risk of heat illness (Cramer and Jay 2016), while even relatively low levels of hyperthermia can impair physical (Périard et al. 2011, 2021; Schlader et al. 2011a) and cognitive performance (Hancock et al. 2007; Schlader et al. 2015a, 2022). Additionally, increased perceptions of warmth and thermal discomfort reduce the ability to successfully carry out complex cognitive tasks (Gaoua et al. 2012). Together, increasing hyperthermia and perceptions of warmth and thermal discomfort can diminish work productivity and quality (Flouris et al. 2018; Foster et al. 2021a). These deleterious effects underscore the need for novel in-work cooling strategies to mitigate the harmful effects of hyperthermia.

While technological advancements such as air conditioning can mitigate the adverse effects of heat stress in indoor environments, such advancements are not feasible in traditional outdoor working environments or in large factories and distribution centers (Morris et al. 2020). Because of this, current guidelines often recommend frequent breaks, but these may not always be practical and adherence is often low (Morrissey et al. 2021, 2023). Thus, feasible and cost-effective personalized cooling strategies may better promote the maintenance of productivity and worker safety during heat stress. One strategy that has proven effective is cooling the glabrous skin on the palm of the hands. By harnessing the unique cutaneous physiology of temperature sensitive arterio-venous anastomoses, palmar cooling can attenuate the rise in core temperature during intense heat stress (Grahn et al. 2005, 2012). However, numerous limitations have prevented the widespread adoption of this technology in the workplace. For example, previous palmar cooling studies used bulky stationary units which served for episodic use only, such as during recovery or for use during a break. Furthermore, these palmar cooling devices compromise the dexterity of the hands and upper limbs such that these devices are unlikely to be feasible for use during work. A potential strategy to overcome these limitations is cooling the glabrous skin on the soles of the feet, which possesses a similar cutaneous physiology (Taylor et al. 2014). Foot cooling may be advantageous because it does not compromise upper limb dexterity and there is high potential for the development of cooling insoles that are more likely to be practical and easily adopted in manual labor sectors than those for hand and upper limb cooling.

A recent study examined the effects of immersing the feet in 20 °C water in older adults and saw no attenuation in core temperature rise during resting heat stress (Meade et al. 2024). Similarly, periodic foot submersion in cool water (22 °C) during resting heat stress showed no effect on the rise in core temperature in younger adults (Morris et al. 2019). Whether these findings translate to a physically active state, similar to that occurring in occupational settings remains unknown. Moreover, to our knowledge no studies have examined the effect of foot cooling on cognitive or physical performance during heat stress. However, previous work has demonstrated improvements in thermal comfort during foot cooling, which could improve cognitive and physical performance (Schlader et al. 2011b; Gaoua et al. 2012). Thus, the purpose of this investigation was to examine the effects of continuous glabrous foot cooling on core temperature, cognitive performance, and physical work capacity during simulated occupational heat stress, focusing on some of the demands encountered by construction workers, a group of U.S. workers at disproportionately high risk of heat related maladies (Dong et al. 2019). The primary purpose of this study was to establish effect sizes for the effect of cooling the soles of the feet on core temperature (primary outcome), cognitive performance (secondary outcome), and physical work capacity (tertiary outcome) in young healthy adults during simulated occupational heat stress. As such, this pilot study tested the hypothesis that cooling the soles of the feet during simulated occupational heat stress will attenuate the rise in core temperature during steady-state physical work, alleviate heat stress induced cognitive performance deficits, and attenuate declines in physical work capacity.

## Methods

### Experimental design

**Fig. 1:**
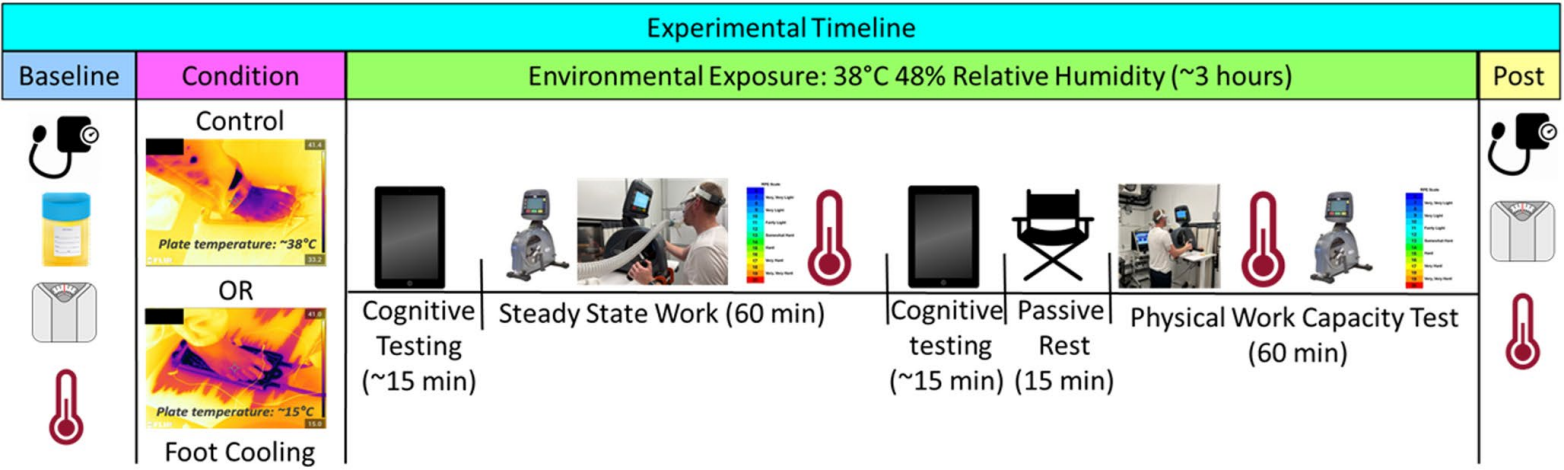
Experimental visit timeline

Before any study activities commenced, this study was approved by the Institutional Review Board at Indiana University (IRB# 16006) and was subsequently performed in accordance with the standards set by the latest revision of the Declaration of Helsinki, except for the registration in a database. This study utilized a block randomized, crossover, balanced design. Participants reported to the laboratory on three separate occasions, with at least 2 days between experimental visits. Visit 1 included consenting, screening, familiarization, and workload determination, the latter of which informed procedures for visits 2 and 3. Visits 2 and 3 were experimental trials, with each trial consisting of 60 min of steady-state exercise, two bouts of cognitive testing, and a 60-minute physical work capacity test (PWCT, Fig. 1). Prior to participation, all participants were informed of the experimental procedures and risks and provided written informed consent.

### Participants

The purpose of this pilot study was to determine the effect sizes for the effect of foot cooling on the changes in core temperature (primary outcome), cognitive performance (secondary outcome), and physical work capacity (tertiary outcome) during simulated occupational heat stress. These effect sizes could then be utilized to design larger trials examining the efficacy of foot cooling in occupational settings. To accomplish this primary objective, we a priori established that we would study a convenience sample size of 12 participants. While the purpose of this study was not to establish statistical significance, it was predicted a priori (G*Power 3.1.9.7) that this sample size would enable the identification of a significant effect of foot cooling on the dependent variables using a two-tailed paired t-test, α = 0.05, 1 – β = 0.80, if the Cohen’s d_z_ effect size was 0.9 or larger. This effect size was considered reasonable based on our previous work demonstrating Cohen’s d_z_ effect sizes between 0.55 (Schlader et al. 2016a) and 1.2 (Chapman et al. 2020) for determining the efficacy of body cooling during simulated occupational heat stress on attenuating the rise in core temperature (i.e., the primary outcome of this pilot study).

Twelve healthy adults (5 women) participated in this study. Participant characteristics were age: 25 ± 6 years, height: 173 ± 11 cm, mass: 70.5 ± 13.2 kg, body mass index: 24.8 ± 3.5 kg/m^2^, body surface area: 1.9 ± 0.2 m^2^. Participants self-reported being moderately active, nontobacco users, and free from any cancer, cardiovascular, colorectal, and hematological diseases/disorders. Participants also did not take any medications that had direct effects on the cardiovascular system. Women self-reported to be normally menstruating and were confirmed not to be pregnant via urine pregnancy test. Four women participants were using contraceptives. We did not control for menstrual cycle in this study as manual labor often takes place throughout the menstrual cycle.

### Instrumentation and measurements

Body height and mass were measured using a stadiometer (Holtain Limited, Seritex, Wales, UK) and scale (Sauter, Baligen, Germany). Urine samples were used to obtain urine specific gravity (USG) via refractometer (Atago, Tokyo, Japan) to ensure participants were well hydrated pre-trial (USG ≤ 1.025). A urine dipstick pregnancy test (human chorionic gonadotropin urine test strip, Clinical Guard, Atlanta, GA, USA) was used to confirm a negative pregnancy test for women. Core temperature was measured via a single-use thermistor (Datex Ohmeda Inc., GE HealthCare, Chicago, Illinois, USA) inserted 10 cm into the rectum. Weighted mean skin temperature was calculated from four wireless temperature sensors (iButtons, Maxim Inc. Columbia, MD) secured to the skin on the chest, triceps, quadriceps, and calf on the right side of the body (Ramanathan 1964). Both feet were instrumented with fine wire thermocouple probes (Omega Engineering Inc., Norwalk, Ct, USA) placed on the plantar side of the big toes at the proximal phalange and the medial arch of the foot. These thermocouple probe locations were chosen to provide an unweighted glabrous skin mean foot temperature. Heart rate was measured via a chest worn heart rate strap (Polar Electro, Kempele, Finland) and is presented as both absolute and as a percentage of age-predicted heart rate maximum (Tanaka et al. 2001). Blood pressure was measured at the right brachium via automated blood pressure cuff (Omron 3 series, Kyoto, Japan) and mean arterial pressure was calculated as diastolic pressure plus 1/3 x pulse pressure. Absolute oxygen uptake (VO_2_) was measured via indirect calorimetry involving a facemask equipped with a one-way valve and a metabolic cart (ParvoMedics, Salt Lake City, Utah). Physical work involved arm ergometry (HealthCare International, Langley, WA). Participants were asked to rate their thermal perceptions via questionnaires every 10 min. Participants gauged body and foot thermal comfort on a scale of 1 (comfortable) to 4 (very uncomfortable), and body and foot pleasantness on a scale of -3.0 (very unpleasant) to + 3 (very pleasant), with 0 being neutral or neither pleasant nor unpleasant. Participants also rated whole body thermal sensation on a scale from 1 (cold) to 7 (hot), skin wetness on a scale of -3 (very dry) to + 3 (very wet) with 0 being neither wet nor dry, and sweating perceptions on a scale of 0 (not sweating) to 10 (most sweating ever).

Cognitive performance testing was performed with a tablet loaded with the Defense Automated Neurobehavioral Assessment (DANA) software (DANA Brain Vital, Silver Spring, MD) (Lathan et al. 2013). At each of the two cognitive performance assessment periods, five distinct cognitive tests were completed in a block randomized order, which consisted of the Go/No-Go, Code Substitution, Spatial Processing, Memory Search, and Match to Sample. The Go/No-Go task assesses impulsivity and ability to sustain attention. The Code Substitution task assesses short-term recall, learning skills, and visual scanning abilities, by assigning a symbol with a number that the participant must recall during the testing. The Spatial Processing task assesses spatial processing by showing a pair of bar histograms with 1 histogram being rotated 90º and the participant must determine if the pair is the same. The Memory Search task assesses short-term memory, via having the participant memorize 5 letters and then trying to recall them as a stream of letters is shown one at a time on the screen. The Match to Sample task assesses working memory, attention, and visuospatial discrimination, by having the participants complete rounds of memorizing a 4 by 4 checkered board which disappears and then reappears with a distractor board and the participant must then select the correct match. All these variables were assessed via a combination of accuracy and speed at which the task was completed, which determined the cognitive efficiency score for each assessment. The cognitive efficiency score was used to assess differences between conditions. More information about these tests can be found on DANA Brain Vital website (https://danabrainvital.com/). DANA assessments were performed while standing, as orthostasis induces a greater circulatory stressor than a seated position (Sjöstrand 1953), with the tablet on a stationary pole set at a standardize height and distance for individual participants to ensure comfort and consistency between trials and tests.

### Visit 1

Visit 1 involved consenting, screening, and familiarization with study procedures. After informed consent was obtained, participants completed a health history questionnaire, a Physical Activity Readiness Questionnaire (PAR-Q) (Warburton et al. 2019), the International Physical Activity Questionnaire (IPAQ) (Craig et al. 2003), and the Montreal Cognitive Assessment (MoCA) (Nasreddine et al. 2005). Participants were required to score 26 or better on the MoCA test to ensure all participants had normal cognitive function. Then height and weight were obtained. Participants then completed a familiarization session with the DANA and a familiarization session with the arm ergometer. Then participants completed a workload determination protocol in which the resistance level and revolutions per minute were adjusted to maintain an absolute VO_2_ of 0.50 L/min. This external workload was used as the workload for the steady-state exercise during Visits 2 and 3. A workload eliciting a VO_2_ of 0.5 L/min was chosen because this is the average VO_2_ observed in construction workers on the jobsite in the heat (Wong et al. 2014), and through preliminary testing was determined to be an intensity able to be completed via arm ergometry for 60 min. Participants were then familiarized with the 6 to 20 Borg Ratings of Perceived Exertion (RPE) scale (Borg and Dahlstrom 1962) and practiced working at a constant intensity that corresponded to a 13 (“somewhat hard”), which was used for the PWCT (detailed below) during Visits 2 and 3. An RPE of 13 was chosen because it is the average RPE in construction workers over a workday (Chan et al. 2012; Wong et al. 2014).

### Visits 2 and 3: experimental trials

Participants were asked to refrain from strenuous exercise, caffeine consumption, and alcohol consumption for 12 h and food for 2 h prior to arrival at the laboratory. Additionally, participants were instructed to consume a volume of water equal to 0.5% of their body weight two hours prior to arrival at the laboratory. Upon arrival to the laboratory, a urine sample was collected for measurement of urine specific gravity and to confirm a negative pregnancy test for women participants. Then participants obtained a nude body mass, which was followed by instrumentation for core temperature, heart rate, and skin temperature. Ten minutes of seated rest preceded baseline measurements of blood pressure, heart rate, and core temperature. After baseline measurements were collected, participants entered the environmental chamber set to 38.7 ± 0.7 ℃ and 46 ± 9% relative humidity. These conditions elicit a wet bulb globe temperature (WBGT) of ~ 31.75 ℃. This WBGT was chosen because it mimics the hot conditions observed during a study of construction workers conducted in Hong Kong (Wong et al. 2014), while this WBGT also represents conditions similar to that encountered by manual laborers in the U.S. (Chapman et al. 2021), including construction workers (Specht et al. 2025a, b).

Upon entry into the environmental chamber, participants had their feet instrumented to record foot temperatures. Following instrumentation, participants completed baseline cognitive testing. After completing the cognitive testing, the participants stepped onto prototype aluminum cooling plates imbedded with copper tubing (Hi-Contact 6-Pass Cold Plate, Boyd Corp., Boca Raton, FL, USA, Fig. 1) with their bare feet, such that the soles of their feet were in direct contact with the surface of the plates. Participants completed one trial when the cooling plates were perfused with 8.6 ± 6.3 ℃ water (Foot Cooling) and in the other trial the plates were not perfused with any water (Control). During the Foot Cooling trial, the resulting plate surface temperature was 19.5 ± 8.9 ℃. This temperature was chosen as it is consistent with the surface temperature utilized in previous studies documenting the effectiveness of palmar cooling (Grahn et al. 2005). During the Control trial, the plate surface temperature was 36.8 ± 0.5 ℃. The order of the Foot Cooling and Control trials was selected in a block randomized manner, which ensured counterbalancing.

Before the start of the 60 min steady-state exercise, participants completed 5 min of standing rest on the plates. During this time perception questionnaires and blood pressure measurements were taken and baseline metabolic data were collected. Participants then started upper arm ergometry steady-state exercise at the predetermined external workload corresponding to a VO_2_ of 0.50 L/min. During the steady-state exercise, metabolic data were collected every 15 min, while perceptions and blood pressure measurements were collected every 10 min. Blood pressure measurements required the participant to briefly (~ 30 s) stop the upper body ergometry exercise. The need to regularly monitor blood pressure was deemed necessary given the increased likelihood of orthostatic hypotension and syncope during heat exposure while in the standing position (Schlader et al. 2016b). Additionally, participants were asked to gently sway throughout the entirety of the standing portions of the study to reduce venous pooling in the legs.

After completing the steady-state exercise, participants completed another bout of cognitive testing while standing on the plates. After cognitive testing, participants completed 15 min of seated rest followed by another 5-minute standing rest period during which participants feet remained firmly on the plates. Participants then started the 60-minute PWCT. The employed PWCT was modified from that employed by Foster et al. (Foster et al. 2021b), which was developed to quantify the impact of heat stress on human physical work capacity. The original model required a continuous adjustment of work output so that a heart rate of 130 bpm is always achieved (Foster et al. 2021b). This heart rate reflects the maximal acceptable cardiovascular strain in occupational settings permitting self-pacing (Foster et al. 2021b). In the present study, a fixed heart rate of 130 bpm could not be utilized because in preliminary testing it was found that heart rate regularly exceeded this level with standing alone and that the addition of upper body work, which leads to higher heart rates compared to lower body exercise (Vokac et al. 1975), led to the cessation of exercise after very little time independent of any cooling. As a result, this study employed a perceptually mediated PWCT, which is consistent with the focus on self-paced work (Schlader et al. 2011b). In this modified PWCT, participants were instructed to exercise at a work rate that could be described as “somewhat hard” or a rating of 13 on the Borg RPE scale. To maintain a constant RPE of 13, participants were asked their perceived RPE every minute and they could voluntarily adjust the cadence or the resistance of the ergometer. Additional thermal perception questionnaires were asked every 10 min, during the 60 min PWCT. Heart rate, core temperature, foot temperatures, skin temperatures, plate temperatures, power output, cadence, and resistance levels were continually recorded throughout the experimental trial.

Upon completion of the PWCT, the thermocouple probes were removed from the participants feet and participants exited the environmental chamber. Upon exiting the environmental chamber, a final core temperature and blood pressure were recorded. Participants were de-instrumented, and a nude body mass was measured. Participants were then free to leave the laboratory.

### Data analysis

Prior to analyses, data were checked for the necessary assumptions for paired t-test and repeated measures linear mixed models. Data normality and the residuals of the linear mixed models were determined to be normally distributed. Consistent with the testing of our hypothesis, core temperature, mean skin temperature, mean foot temperature, perception questionnaires, and mean power output data were analyzed using 2-way repeated-measures linear mixed models (time, condition: control or foot cooling, and the interaction between time x condition), for the individual components of 1-hour steady-state exercise, DANA assessments’ cognitive efficiency scores, rest interval (end of steady-state exercise/DANA to the start of PWC test), and 1-hour PWCT. If a main effect or interaction was observed, post hoc tests using Sidak’s methods for multiple comparisons were conducted. For pairwise comparisons Cohen’s d_z_ effect sizes are reported, including the primary outcome of change in core temperature, secondary outcome of the change in cognitive function efficiency scores, and the tertiary outcome of the change in physical work capacity average power. Additionally, paired t-tests were used to examine changes in body mass from pre- to postexposure and changes in core temperature from pre- to post- 1-hour steady-state exercise, the rest interval, and the 1-hour PWCT. Mean foot temperatures for rest interval were only record for 6 individuals for both conditions, as reapplying thermocouples to the feet between steady-state work and the physical work capacity test occurred at this time for various participants who pulled them off when rotating to complete the DANA task and/or moving to start the sitting rest. Percent changes in body mass from pre- to post-exposure provided an index of changes in total body water. All data were analyzed via GraphPad Prism Version 10.2.0. A priori statistical significance was set at *p* ≤ 0.05, and actual p-values are reported where possible. Data reported as mean ± standard deviation and with individual values.

## Results

### Steady-state exercise

**Fig. 2:**
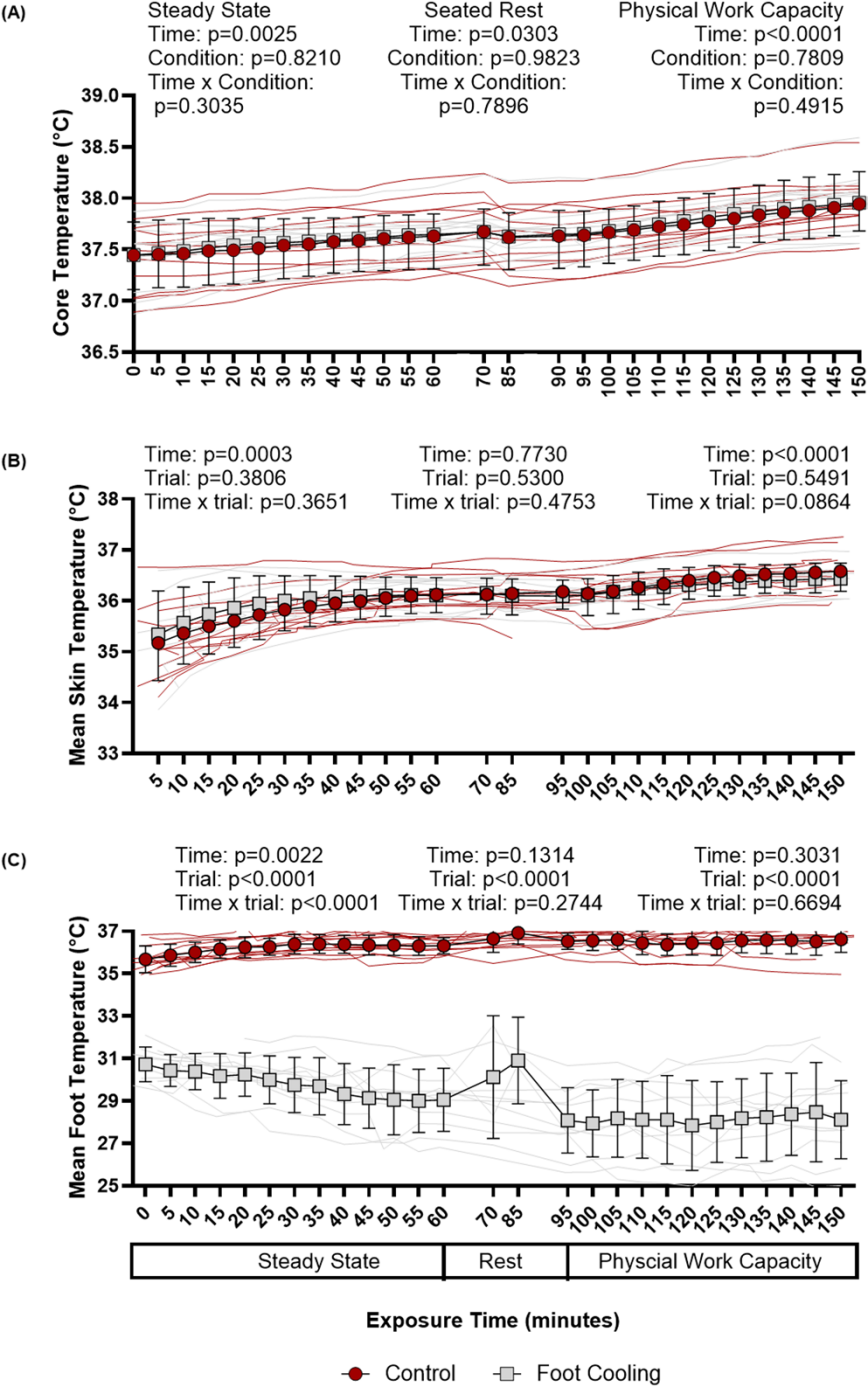
Core (rectal) temperature (A), mean skin temperature (B), and mean foot temperature (C) responses during environmental exposure. Mean foot temperatures for rest interval were only recorded for 6 individuals for both conditions, as reapplying thermocouples to the feet between steady-state work and the physical work capacity test occurred at this time for various participants who pulled them off when rotating to complete DANA task and/or moving to start the sitting rest. P-values presented from linear mixed models for main effects and interaction for steady-state, rest, and physical work capacity tests. Data are presented as group averages with SD error bars

**Fig. 3:**
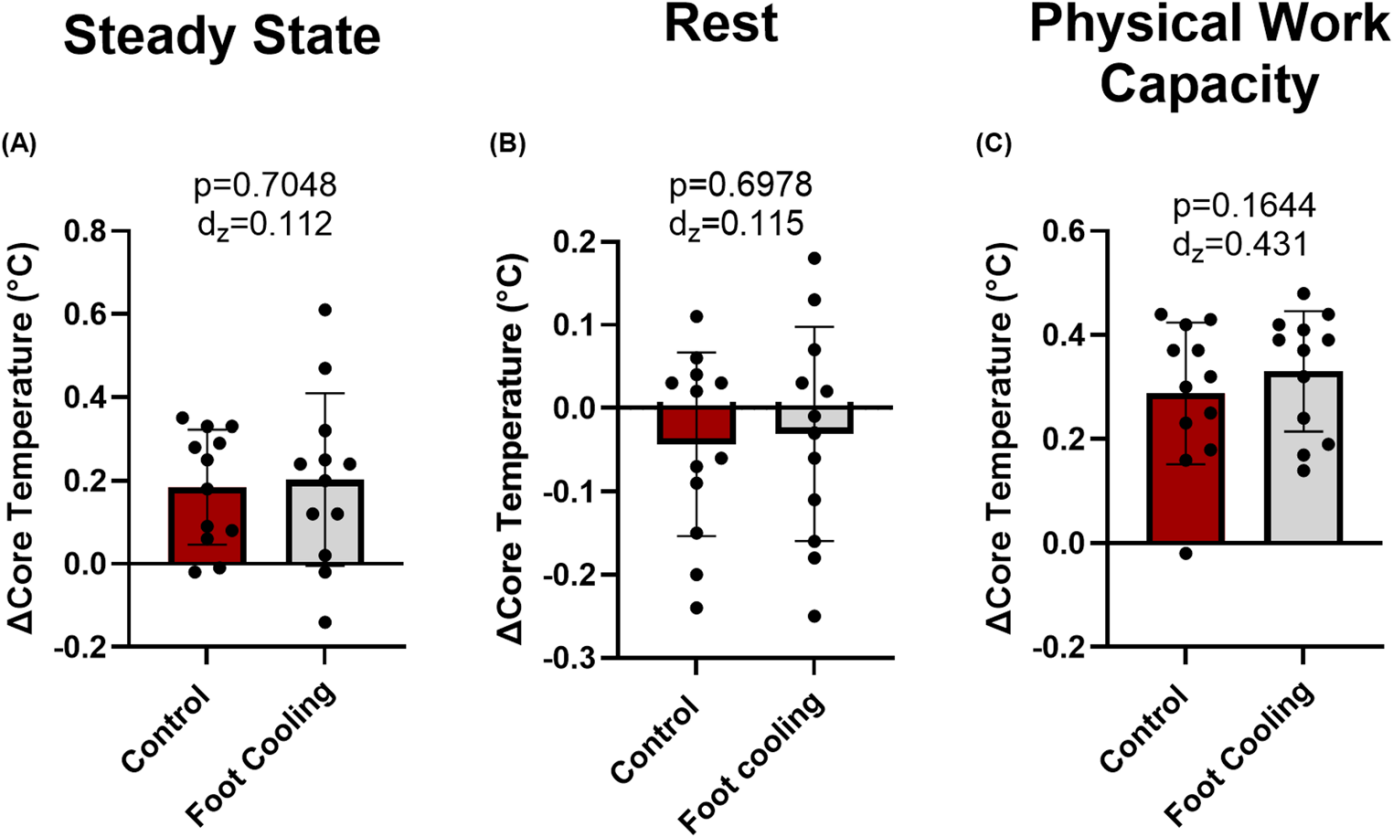
Change in core temperature from start to end of steady-state (A), rest (B), and physical work capacity (C) tests. P-values for paired t-tests are provided along with Cohen’s d_z_ effect sizes. Data are presented as group averages with SD error bars. Black circles represent individual values

**Fig. 4:**
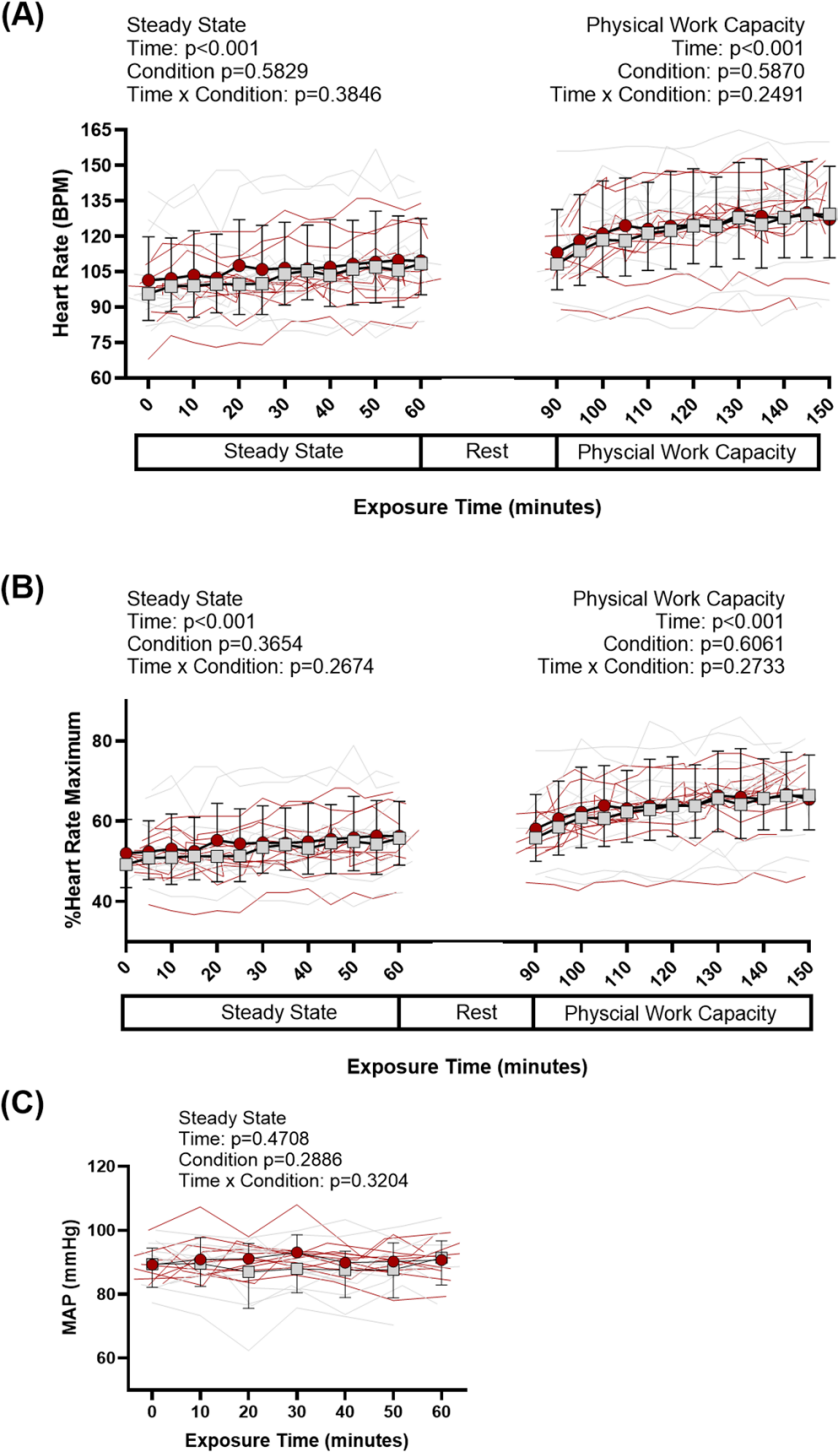
Heart Rate (A) and % Heart Rate Maximum (B) responses across environmental exposure. Mean Arterial Pressure (MAP, C) response during steady-state exercise. P-values presented from linear mixed models for main effects and interaction for steady-state, rest, and physical work capacity tests. Data are presented as group averages with SD error bars

During steady-state exercise, there was no difference between foot cooling and the control conditions for VO_2_ (*p* = 0.544) with an average VO_2_ of 0.5 ± 0.0 L/min (Foot Cooling) and 0.5 ± 0.1 L/min (Control). Core temperature increased from the start of steady-state exercise to the end (main effect of time: *p* < 0.003), but there was no difference in core temperature between conditions (main effect of condition: *p* = 0.821, Fig. 2A) or in the change in core temperature during steady-state exercise (paired t-test: *p* = 0.705, Fig. 3A). Mean skin temperature increased from the start of steady-state exercise to the end (main effect of time: *p* < 0.001) but mean skin temperature did not differ between conditions (main effect of condition: *p* = 0.381, Fig. 2B). Mean foot temperatures during steady-state exercise were lower during foot cooling compared to the control condition (main effect of condition: *p* ≤ 0.002, Fig. 2C), with final foot temperatures of 29.5 ± 2.1 ℃ and 36.3 ± 0.4 ℃, respectively. Heart rate increased during steady-state exercise (main effect of time: *p* < 0.001, Fig. 4A) but did not differ between conditions (main effect of condition: *p* = 0.583, Fig. 4A), with average heart rates 106 ± 3 bpm (foot cooling) and 103 ± 4 bpm (control). Mean arterial pressure did not differ over time (main effect of time: *p* = 0.471) or between conditions (main effect of condition: *p* = 0.288), with average mean arterial pressures of 88 ± 2 mmHg (foot cooling) and 91 ± 1 mmHg (control).

**Fig. 5:**
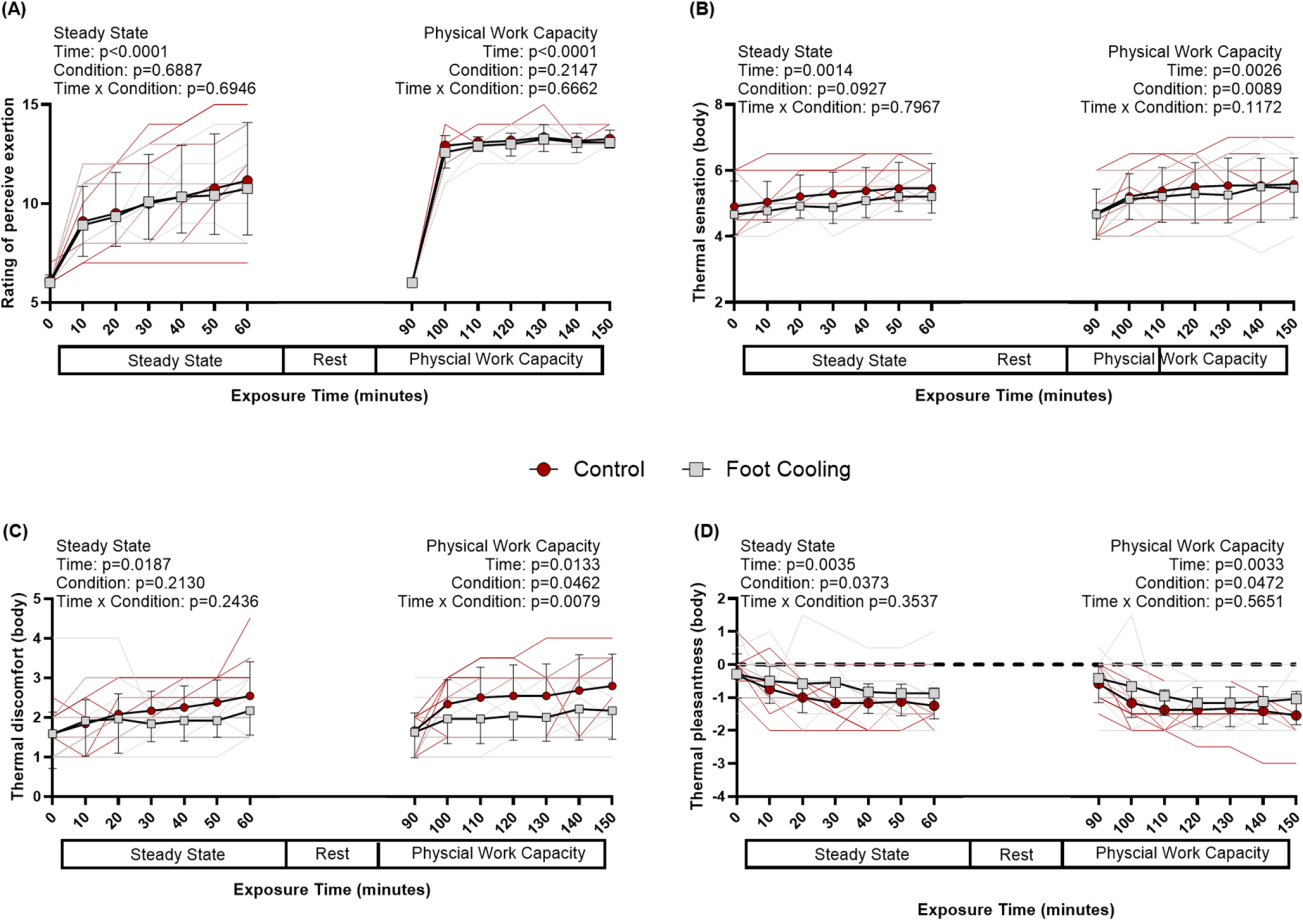
Perceptual responses for rating of perceived exertion (A), whole body thermal sensation (B), whole body thermal discomfort (C), whole body thermal pleasantness (D), during steady-state work and physical work capacity tests. Timepoints 0 and 90 are participant perceptions prior to the start of work for both tests. P-values presented from linear mixed models for main effects and interaction for steady-state and physical work capacity tests. Data are presented as group averages with SD error bars

Participants demonstrated an increase in RPE, and whole-body thermal sensation, thermal discomfort, and thermal pleasantness (main effect of time: *p* < 0.018, Fig. 5) during steady-state exercise. Additionally, the perception of skin wetness and sweating during steady-state exercise increased (main effect of time: *p* < 0.001) with ratings increasing from 0.6 ± 0.1 a.u. to 1.5 ± 0.3 a.u. and 2.1 ± 0.3 a.u. to 4.8 ± 0.5 a.u. when collapsed across conditions, respectively. Perceptions of feet thermal discomfort and pleasantness had interaction effects (*p* = 0.047 and *p* = 0.031, respectively) during steady-state exercise, but no significant time, condition, or pairwise comparisons. The final mean rating for feet thermal discomfort was 2.3 ± 0.1 a.u. and for feet thermal pleasantness was − 1.2 ± 0.1 a.u. While there were no condition effects for RPE, or whole-body thermal sensation or thermal discomfort, whole-body thermal pleasantness was reduced during foot cooling (main effect of condition: *p* = 0.037, Fig. 5D). Similarly, perception of skin wetness was also decreased during foot cooling condition (main effect of condition: *p* = 0.039), with a final perceived wetness of 1.7 ± 0.6 a.u. for the control condition and 1.3 ± 0.5 a.u. for the foot cooling condition.

### Cognitive performance

**Fig. 6:**
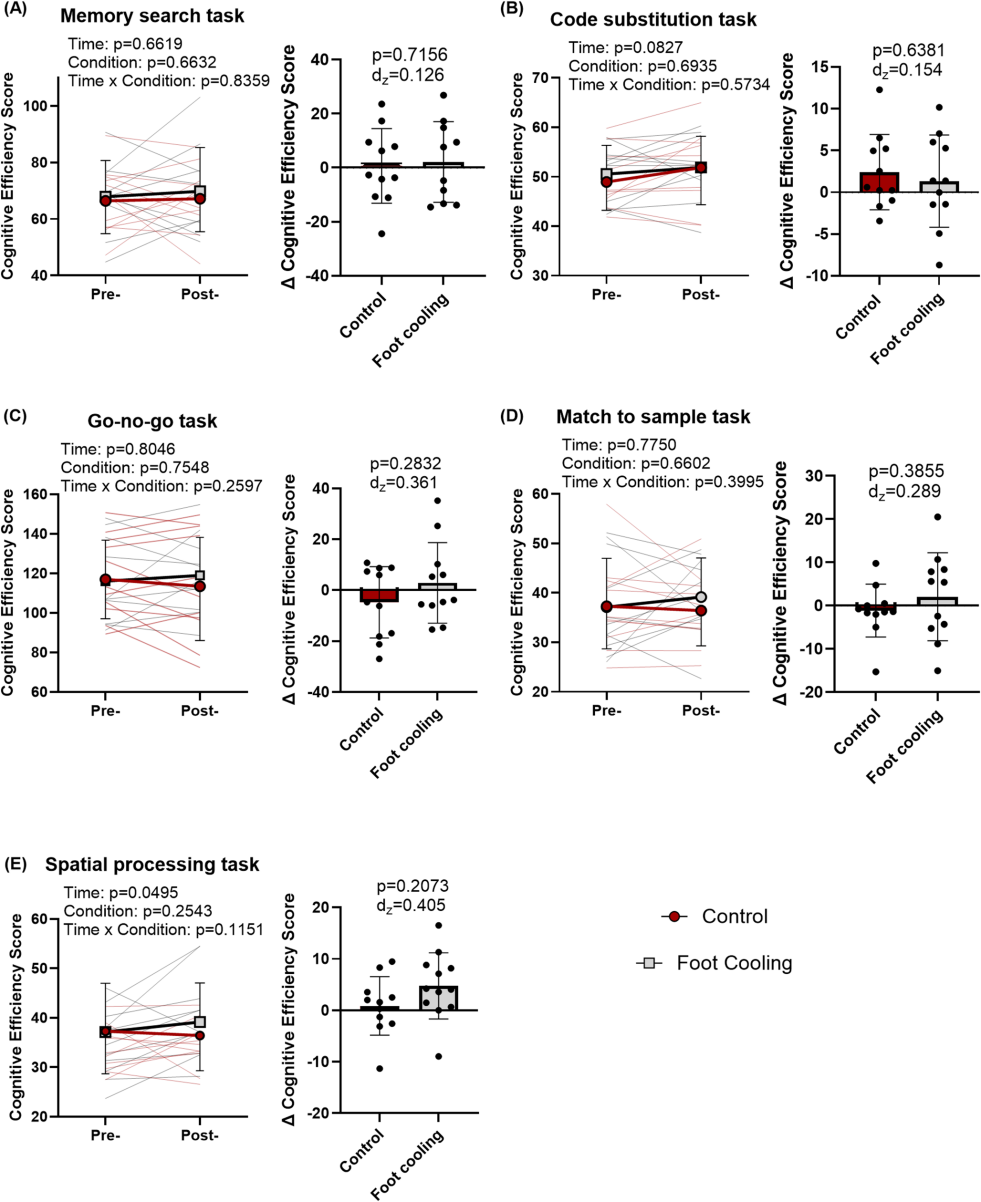
Cognitive efficiency scores from the Defense Automated Neurobehavior Assessment - Brief for Memory search task (A), Code substitution task (B), Go-no-Go task (C), Match to sample task (D), spatial processing task (E) pre and post steady-state exercise and change in cognitive efficiency score between conditions. P-values presented from linear mixed models for main effects and interaction, and p-values and Cohen’s d_z_ presented for pairwise comparison for change (Δ) in cognitive efficiency scores. Data are presented as group averages with SD error bars

The pre to post steady-state exercise DANA assessments showed no time, condition, or interaction effects on cognitive efficiency for the cognitive assessments of go-no-go task, code substitution, memory search, and match to sample (*p* ≥ 0.083, Fig. 6). However, for spatial processing there was an increase in cognitive efficiency following steady-state exercise (main effect of time: *p* = 0.049) with no difference between the conditions (*p* = 0.254) or the interaction (*p* = 0.115, Fig. 6E).

### Rest interval

During the 15-minute seated rest interval, core temperature decreased (*p* = 0.030), but there was no difference in core temperature between conditions (*p* = 0.982, Fig. 2A) or in the change in core temperature between conditions (*p* = 0.790). Mean skin temperature did not change from the start of rest to the end (*p* < 0.773) and there was no difference in skin temperature between conditions (*p* = 0.530, Fig. 2B). Foot temperatures (*n* = 6) during rest were lower during foot cooling compared to the control condition (*p* < 0.001, Fig. 2C), with mean foot temperatures of 30.5 ± 0.38 ℃ and 36.9 ± 0.04 ℃, respectively.

### Physical work capacity test

**Fig. 7:**
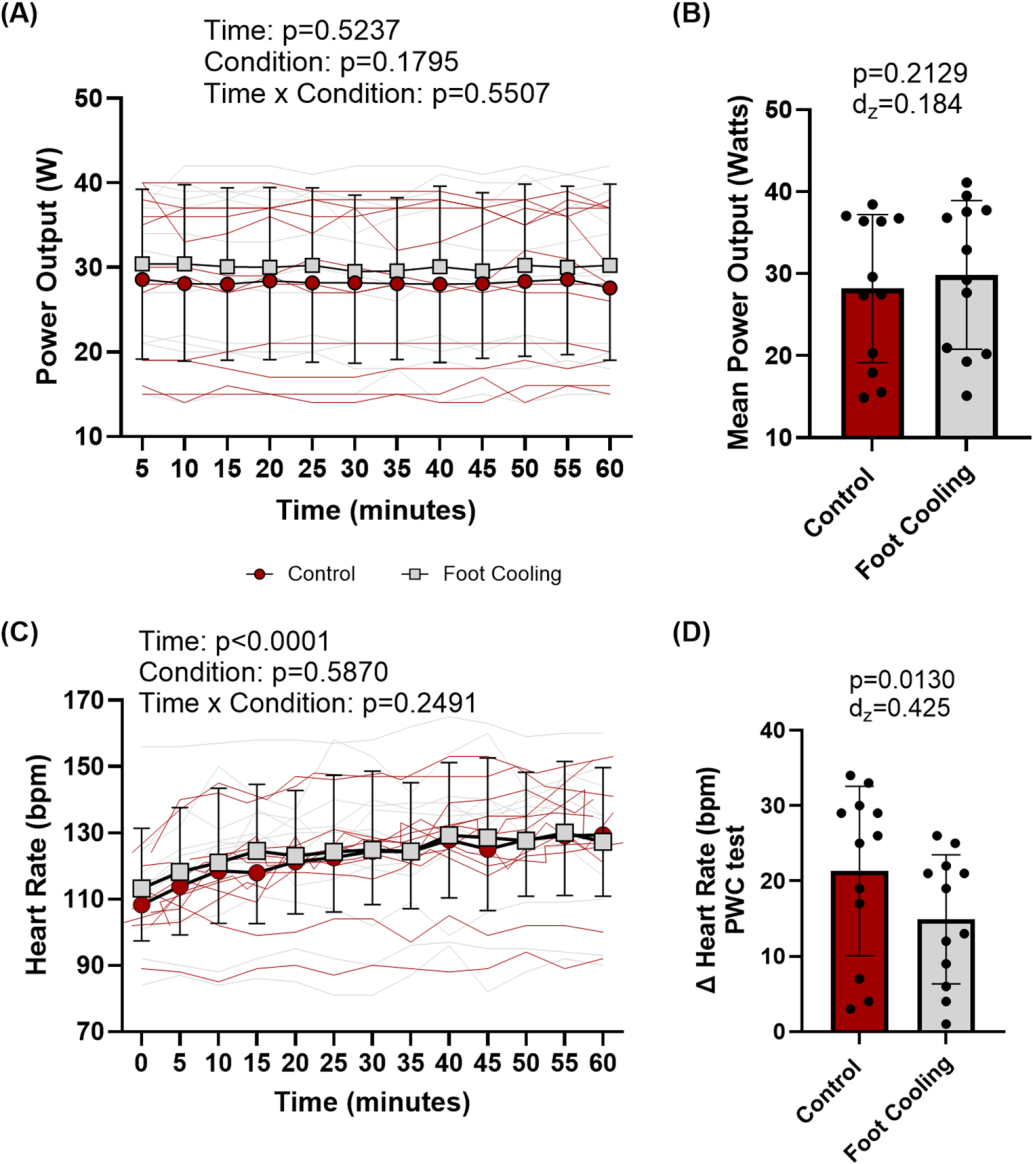
Average power output response during physical work capacity test. (A) 5-minute averages of power output during physical work capacity tests, with p-values presented from linear mixed models for main effects and interaction. (B) Mean power output of during physical work capacity tests, with p-value for paired t-tests and Cohen’s d (d_z_) effect size. (C) Heart rate data every 5 min during the physical work capacity test, with p-values presented from linear mixed models for main effects and interaction. (D) Change in heart rate from pre to post physical work capacity test, with p-value for paired t-tests and Cohen’s d_z_ effect size. Data are presented as group averages with SD error bars. Black circles represent individual values

During the PWCT, there was no difference between foot cooling and the control conditions for mean power output (main effect of condition: *p* = 0.180, Fig. 7B), with final mean power outputs of 30 ± 10 and 28 ± 9 W, respectively. Core temperature increased from the start of PWCT to the end (main effect of time: *p* < 0.001), but there was no difference in core temperature between conditions (main effect of condition: *p* = 0.781, Fig. 2A) or in the change in core temperature (paired t-test: *p* = 0.164, Fig. 3C). Mean skin temperatures increased from the start of the PWCT to the end (main effect of time: *p* < 0.001), but there was no difference between conditions (main effect of condition: *p* = 0.549, Fig. 2B). Foot temperatures during the PWCT were lower during foot cooling compared to the control condition (*p* < 0.001), with final foot temperatures of 28.12 ± 1.84 ℃ and 36.62 ± 0.62 ℃, respectively. Heart rate increased during PWCT (main effect of time: *p* < 0.001, Fig. 7C) but did not differ between conditions (main effect of condition: *p* = 0.587, Fig. 7C). Foot cooling (15 ± 9 bpm) reduced increases of heart rate from pre- to post- PWCT compared to the control (21 ± 11 bpm, *p* = 0.013, Fig. 7D).

RPE increased from rest to starting the PWCT (main effect of time: *p* < 0.001, Fig. 5A), but then there was no difference at any timepoint after the start of the PWCT (all post-hoc tests: *p* > 0.720). Whole-body thermal sensation, thermal discomfort, and thermal unpleasantness increased (main effect of time: *p* < 0.013, Fig. 5) during the PWCT. However, foot cooling trials increased significantly less than control trials for all three perceptions (main effect of condition: *p* < 0.047, Fig. 5). Perceptions of thermal discomfort of the feet were less during the foot cooling trials (main effect of condition: *p* = 0.046) and discomfort increased with trial duration for the control trial (main effect of time: *p* = 0.013, and the interaction: *p* = 0.008). The final mean rating for feet thermal discomfort was 1.9 ± 0.8 a.u. for the foot cooling trial and 2.7 ± 0.9 a.u. for the control trial. Perceptions of thermal pleasantness of the feet had an interaction effect (*p* = 0.042) during the PWCT, with decreases of thermal pleasantness in control trials compared to foot cooling trials at 20 (– 1.5 ± 0.9; – 0.8 ± 1.0 a.u.) and 60 (– 1.5 ± 0.9; – 0.8 ± 0.9 a.u.) minutes with mean differences of – 0.6 ± 0.2 and − 0.6 ± 0.2 a.u., at the respective timepoints. Additionally, the perception of skin wetness and sweating increased from the start to end of the PWCT (main effect of time: *p* < 0.001) in mean rating from 0.9 ± 0.2 a.u. to 1.8 ± 0.2 a.u. and 2.5 ± 0.4 a.u. to 5.4 ± 0.8 a.u. when collapsed across conditions, respectively. Perceptions of skin wetness and sweating were not different between trials (main effect of condition: *p* > 0.086).

### Entire duration of exposure

Nude body weight was decreased from pre- to post-exposure in the control and cooling conditions (control: 1.26 ± 0.17%, foot cooling: 1.03 ± 0.56%, *p* < 0.001), but there was no difference between conditions (*p* = 0.711). Total body sweat rate was not different between conditions (paired t-test: *p* = 0.460) with average sweat rates of 0.32 ± 0.08 L/hr for the foot cooling and 0.34 ± 0.06 L/hr for control trials.

## Discussion

We tested the hypothesis that cooling the soles of the feet would attenuate the rise in core temperature during steady-state exercise, alleviate heat induced cognitive performance deficits, and augment physical work capacity in young healthy adults during simulated occupational heat stress. We observed that simulated occupational heat stress involving only upper body work increased core temperature, but this elevation was not altered by the employed method of foot cooling (Figs. 2 and 3). Additionally, cognitive performance across a range of domains (Fig. 6) and physical work capacity (Fig. 7) were not affected by foot cooling. These results do not support our hypothesis, and are consistent with previous work in younger (Morris et al. 2019) and older adults (Meade et al. 2024) showing that lower limb and foot cooling do not attenuate the rise in core temperature during passive heat stress. It should be noted, however, that the main goal of this work was to establish effect sizes for future experiments investigating the effects of foot cooling at attenuating the rise in core temperature (primary outcome), alleviating heat induced cognitive performance deficits (secondary outcome) and improving physical work capacity (tertiary outcome) during occupational heat stress. To that end, the reported effect sizes will inform future work related to foot cooling and the potential benefits. This work adds to the literature by providing effect sizes for future investigations, while also demonstrating that foot cooling during relatively mild heat stress combined with upper body physical work over an ~ 3-hour period is unlikely to alter cognitive function or physical work capacity.

### Primary outcome—change in core temperature

Over the duration of the experiment, we observed an increase in core temperature during each hour of work. However, foot cooling had no discernable effect on core temperature (Figs. 2 and 3) during the relatively mild occupational heat stress simulation employed herein. This finding aligns with previous work examining the efficacy of foot cooling on core temperature during passive heat stress (Morris et al. 2019; Meade et al. 2024). In explanation of our findings, it is possible that the magnitude of simulated occupational heat stress, and the subsequent rise in core temperature, was too low to observe the efficacy of foot cooling. For example, the workload could have been too light, the severity of environmental heat exposure too cool, and/or the duration of heat stress too short to observe any potential benefits, or a combination of all these factors (Fig. 3). This is supported by the findings of Morris et al. who also saw a very modest rise in core temperature during two hours of passive heat stress and also failed to demonstrate foot cooling as an effective cooling modality (Morris et al. 2019). Previous work demonstrating the beneficial effects of continuous palmer cooling on core temperature had participants complete a more strenuous exercise bouts in the heat that resulted in a core temperature rise of more than 2.0 °C (Grahn et al. 2005). Similarly, another investigation using post-exercise cooling identified faster core temperature cooling rates with palm cooling compared to passive cooling when participants had an average rise in core temperature of over 2.0 °C (Adams et al. 2016). In the current investigation, in which the rise in core temperature was only ~ 0.5 °C, foot cooling during physical work or passive rest (Fig. 2) did not affect core temperature. Thus, compared to the current investigation the magnitude of heat strain was more pronounced in the previously mentioned palm cooling studies. That said, a strength of our study was that it was designed a priori to simulate some of the metabolic and environmental stressors encountered by construction workers. A limitation of our approach, however, was the duration of the simulation (~ 3 h total). This was deemed to be a practical limit to conducting continuous upper body work. Therefore, we encourage future studies to utilize longer duration heat stress scenarios when examining the efficacy of cooling modalities with application to occupational settings.

A notable difference between our study and the palm cooling literature is the use of a negative pressure to draw blood into the hand. Thus, it is possible that the observed ineffectiveness of foot cooling at attenuating the rise in core temperature, could be that the reduction in local skin temperature caused vasoconstriction and reduced local blood flow through the feet. That said, while there might be slightly lower than maximal blood flow to the foot, previous work has shown with a hyperthermic core temperature (38.5 ℃) and a cool local skin temperature of the foot (25.1 ℃), the average blood flow through the foot would be increased (6.1 ml/100 ml*min) compared to thermoneutral core temperature (36.9 ℃) with the same local skin temperature (blood flow of foot = 2.2 ml/100 ml*min) (Taylor et al. 2014). Thus, local cooling-induced vasoconstriction does not completely eliminate the influence of increased core temperature on blood flow to the feet (Taylor et al. 2014). Moreover, we estimate that both the foot cooling and control conditions (average foot temperatures of 28.6 ℃ and 37.1 ℃, respectively) would have an estimated blood flow through the foot of 6.1–9.3 ml/100 ml*min (Taylor et al. 2014). Thus, foot blood flow is likely increased in both conditions of our experiment with little difference between the conditions. Another thought is that colder plates may have an effect since blood flow through the foot was probably maintained. However, further research is needed to elucidate this possibility. Additionally, the previously mentioned palm cooling studies used a device which utilized negative pressure to pull more blood flow into the hand, which may have maximized blood flow and aided in the core temperature attenuation and cooling (Grahn et al. 2005; Adams et al. 2016). Unfortunately, applying negative pressure to the feet, would increase the risk of syncope due to blood pooling in the lower body, especially if the person was dehydrated (Schlader et al. 2015b). Thus, adding negative pressure to the feet is not feasible for a foot cooling intervention.

### Secondary outcome—change in cognitive performance

The secondary aim of this investigation was to determine if the deleterious effects of mild simulated occupational heat stress on cognitive performance can be mitigated by foot cooling. This investigation demonstrated no effect of mild heat stress on most of the assessed domains of cognitive function following one hour of steady-state upper arm work in the heat, the exception being spatial processing as there was a time effect demonstrating an *improvement* in the cognitive efficiency scores following 60 min of steady-state upper body exercise in the heat (Fig. 6). Our data align with previous work on cognitive performance during passive heat stress that increased core temperature by ~ 1.0 ℃ and demonstrated no effect or a slight augmentation of cognitive function within different domains (Schlader et al. 2015a). In contrast to that passive heat stress work, in the current study core temperature increased by ~ 0.2 ℃ prior to the cognitive assessments and most domains of cognition were unaffected. Thus, the level of hyperthermia during this investigation was likely much lower than that required to elicit measurable reductions in cognitive performance. Therefore, the lack of observed cognitive impairments was likely due to the relatively mild nature of the employed occupational heat stress simulation that, in turn, impaired our ability to identify whether glabrous skin foot cooling was beneficial at restoring cognitive performance. Indeed, similar discrepancies have been reported in the literature such that previous work has shown cognitive benefits, decrements, and no change to cognitive performance during heat stress (Hancock and Vasmatzidis 2003; Taylor et al. 2016; Doohan et al. 2023). The inconsistencies have been based on a variety of factors including domain of assessment, exposure duration, intensity of heat stress, perception, acclimatization, and skill level of a task (Périard et al. 2021).

### Tertiary outcome—change in physical work capacity

While previous studies have demonstrated that physical work capacity is decreased in proportion to the magnitude of hyperthermia (Périard et al. 2011, 2021; Schlader et al. 2011c; Flouris et al. 2018; Foster et al. 2021a), intermittent palm (Grahn et al. 2005, 2012; Kwon et al. 2010) and/or foot cooling (Cai et al. 2021; Wu et al. 2021) has shown improvements in physical performance during non-heat stress conditions (Kwon et al. 2010; Cai et al. 2021; Wu et al. 2021) and during heat stressed conditions (Grahn et al. 2005, 2012). Therefore, the tertiary aim of this investigation was to determine if plantar foot cooling during heat stress influences sustainable self-selected upper body ergometry work rate, as quantified by the power output required to maintain an RPE of 13 during arm ergometry. The results demonstrated no difference in mean power output between conditions (Fig. 7A-B) when performing one hour of work at an average RPE observed during construction work (Wong et al. 2014). The lack of improvement in performance despite lessening whole-body thermal discomfort was likely due to the inability of the foot cooling to meaningfully increase heat loss during mild simulated occupational heat stress. This finding is surprising in light of other studies showing that improving thermal discomfort (independent of differences in heat loss) increased work output during fixed RPE lower-body cycle ergometer exercise (Schlader et al. 2011b). Thus, our findings may be more specific to the relatively mild heat stress conditions employed and relatively small muscle mass engaged via upper body exercise. However, an investigation completed by Ely et al. demonstrated modest elevations in core temperature (similar to this study) can lead to decrements in aerobic (lower-body cycle ergometry) performance. Since both conditions in this study had a similar rise in core temperature and the foot cooling was likely unable to meaningfully improve heat loss, it is possible that performance decrements occurred in both trials without a difference for the 1-hour test. As mentioned previously, however, investigations at comparatively higher core temperatures and for longer work durations may unmask the potential benefits of glabrous skin foot cooling on physical work capacity. Specifically, foot cooling reduced increases of heart rate during the physical work capacity test compared to the control (Fig. 7D), and this effect may provide a benefit by reducing cardiovascular strain, if that trend continues during extended duration scenarios. For example, while not directly measured in this study, localized cooling of the feet may have reduced lower leg and foot blood flow circulation during foot cooling trials, which would have improved venous return thereby reducing the need for a continued elevation in heart rate to sustain the necessary cardiac output to accommodate the enhanced peripheral blood flow during mild heat stress. This alteration could change the onset of cardiovascular drift, which occurs earlier than the limits of heat balance (Cottle et al. 2023), and thus provide a beneficial effect, particularly given that cardiovascular events are often a contributing factor to many heat related deaths (Loughnan et al. 2010). Therefore, future work should investigate the benefits of reduced cardiovascular strain and cardiovascular drift during heat stress and foot cooling.

### Experimental considerations

As this was a pilot study to determine the potential benefits of plantar foot cooling in the context of simulated occupational heat stress, there are several experimental considerations. First, while there was precedent for the chosen environmental conditions (Specht et al. 2025a, b; Wong et al. 2014) and workload (Wong et al. 2014), the resulting occupational heat stress simulation resulted in relatively modest rises in core temperature. Thus, as noted above, future work examining the effect of plantar foot cooling in the context of simulated occupational heat stress should consider more severe heat stress conditions. Similarly, it is worth noting that participants only completed ~ 3 h of work in the heat. This is substantially less than often required in occupational scenarios (e.g., 8–12 h). Thus, completing a longer duration exposure would increase the external validity and applicability to the workplace situation. A second consideration is the stationary nature of arm ergometry, which was required for this study to allow physical work to be completed, while maintaining contact with the cooling plates. Such exercise may simulate factory or assembly line work but fails to incorporate any lower body work. While participants were asked to gently sway the entire time, to prevent syncope, many occupations require lower body work. Importantly, increased locomotion in the lower body could alter blood flow through the plantar region of the foot, as blood flow through the foot changes based on walking speed (Wu et al. 2020). A potential increase in blood flow could aid in the cooling of the blood returning to the body core. Therefore, future studies that involve movement of the lower limbs may provide further insights into potential efficacy of foot cooling and enhance the ecological validity for more occupations. Additionally, this investigation did not control for menstrual cycle, which may alter thermoregulation during exercise in the heat (Giersch et al. 2020). Thus, future studies should consider controlling for the menstrual cycle. However, it should be noted that women regularly perform physical labor in the heat without consideration for their menstrual cycle phase.

## Conclusion

Cooling the plantar surface of the feet using a water infused cooling plate did not attenuate the rise in core body temperature during ~ 3 h of simulated occupational heat stress that included steady-state arm ergometry exercise, cognitive test bouts, 15 min of rest, and a physical work capacity test with arm ergometry. Additionally, cognitive function across domains measured via the DANA-Brief cognitive test battery and performance on a perceptually mediated physical work capacity test were unaltered by foot cooling during heat stress. While these findings suggest that cooling the feet may not mitigate decrements in cognitive or physical performance during upper body work under mild heat strain, it should be noted that this was a pilot study to determine effect sizes for better powered future investigations. To that end, we have established effect sizes for the change in core temperature, cognitive performance and physical performance. Future work should use these effect sizes to inform sample size justification. Future investigations should consider examining foot cooling during more severe and/or prolonged heat stress conditions, particularly those involving lower body work and locomotion.
